# Serum long noncoding RNA FAM83H-AS1 serves as a potential noninvasive diagnostic biomarker for ovarian cancer

**DOI:** 10.1186/s13048-026-01995-1

**Published:** 2026-02-03

**Authors:** Chunying Tian, Hongshuai Sun, Ruiyao Li, Yuanyuan Chen, Ting Li, Xiaoming Su, Xiuyan Yu

**Affiliations:** Department of Clinical Laboratory, Jilin Cancer Hospital, Changchun, 130012 China

**Keywords:** Ovarian cancer, Long noncoding RNA, FAM83H-AS1, Serum, Biomarker

## Abstract

**Background:**

The long noncoding RNA (lncRNA) FAM83H-AS1 plays a critical role in the development and progression of various cancers. This study evaluates the feasibility of serum FAM83H-AS1 as a diagnostic and staging marker for ovarian cancer (OC), addressing its previously unclear diagnostic value.

**Methods:**

Our study included 118 patients diagnosed with OC, 73 individuals with benign ovarian disease (BOD), and 61 healthy controls (HCs). The expression of serum FAM83H-AS1 was quantified via RNA extraction followed by RT-qPCR. The levels of CA125 and HE4 were detected via a chemiluminescence microparticle immunoassay, and the risk of ovarian malignancy algorithm (ROMA) was calculated. Receiver operating characteristic (ROC) curve analysis was used to evaluate the diagnostic effect of FAM83H-AS1. Univariate and multivariate Cox regression analyses were employed to identify independent prognostic factors, while Kaplan–Meier analysis with the log-rank test was used to compare survival differences.

**Results:**

The levels of FAM83H-AS1 were significantly elevated in OC patients compared with those in individuals with BOD and HCs (*P* < 0.001). Additionally, FAM83H-AS1 expression levels were markedly higher in advanced TNM stages and in cases of metastasis. ROC curve analysis demonstrated robust diagnostic performance for FAM83H-AS1 alone (AUC = 0.824), and the combined FAM83H-AS1 + ROMA panel achieved superior accuracy (AUC = 0.909). Stepwise multivariate analysis identified FAM83H-AS1 as an independent diagnostic predictor for OC. A unit increase in this marker was associated with a 6.9% increase in the odds of OC (*P* = 0.030). FAM83H-AS1 levels were positively correlated with TNM stage, lymphatic metastasis, distal metastasis, peritoneal metastasis, and established biomarkers (CA125, HE4, and ROMA; *P* < 0.05). Notably, postoperative FAM83H-AS1 levels were significantly lower in 22 paired patients (*P* < 0.01), confirming its tumor-derived origin. Survival analysis indicated that elevated serum levels of FAM83H-AS1 expression serve as an independent prognostic factor associated with decreased progression-free survival (PFS) (HR = 8.251, *P* = 0.041). However, it failed to exhibit independent prognostic significance for overall survival (OS), as the correlation with OS observed in the univariate analysis was rendered non-significant in the multivariate analysis after adjusting for TNM stage. Kaplan-Meier curves further validated significantly worse survival outcomes for patients categorized in the high-expression group (*P* < 0.05).

**Conclusions:**

This research is the first to indicate that serum FAM83H-AS1 may serve as a novel noninvasive biomarker for the diagnosis of OC. The combination of FAM83H-AS1 and ROMA demonstrated excellent diagnostic efficacy. Serum FAM83H-AS1 is promising for monitoring the stage and progression of OC patients.

**Supplementary Information:**

The online version contains supplementary material available at 10.1186/s13048-026-01995-1.

## Introduction

To date, ovarian cancer (OC) remains one of the major cancers affecting women worldwide, with an incidence rate of 3.4%, ranking eighth among female cancers. Its mortality rate is 4.8%, making it the fifth leading cause of cancer-related fatalities. Over 320,000 women are diagnosed annually, resulting in approximately 200,000 deaths, underscoring the significant threat this disease poses to women’s health and survival [1]. The prognosis for OC patients is poor, with a survival rate of only 30%, primarily due to the insidious early symptoms of OC and the absence of straightforward and accurate early diagnostic methods in clinical practice. This results in nearly 70% of women being diagnosed at a late stage [2]. Current diagnostic modalities for OC include transvaginal ultrasound, computed tomography (CT), and serum tumor markers such as CA125 and HE4 [3]. However, these existing methods have notable limitations, as they lack sufficient specificity and sensitivity for the reliable identification of malignancy, particularly in early detection when other benign gynecological conditions are considered [4, 5]. These challenges highlight the urgent need for more reliable and novel noninvasive diagnostic tools for OC detection.

Long noncoding RNAs (lncRNAs) are a class of noncoding RNAs that do not encode proteins and are typically longer than 200 nucleotides [6]. LncRNAs can influence numerous physiological functions through mechanisms such as transcriptional control, chromatin remodelling, and posttranscriptional regulation [7], thereby promoting the progression of OC via the regulation of signalling pathways [8–10]. LncRNAs are recognized for their critical roles in cancer cell proliferation, invasion, metastasis, immortality, and angiogenesis [11, 12]. Previous reports have documented alterations in the expression of lncRNAs across various cancer types, including OC [13–15]. Notably, prostate cancer antigen 3, a novel noncoding RNA, has been approved by the U.S. FDA as an innovative molecular biomarker for the diagnosis of prostate cancer [16].

Recent studies have indicated that FAM83H antisense RNA 1 (FAM83H-AS1), a newly discovered lncRNA, plays a functional role in various cancer types, particularly lung, breast, bladder, and pancreatic cancers [17–22]. Studies have shown that FAM83H-AS1 is markedly elevated in gastric cancer tissues and cell lines, with higher expression levels observed in patients with greater tumor invasion, greater differentiation, and chemoresistance [23]. Research has confirmed that FAM83H-AS1 is highly expressed in OC tissues compared with that in peritumoral tissue, with expression levels varying across different stages of the disease. Increased levels of FAM83H-AS1 are associated with poorer outcomes for OC patients [9]. To date, research on the expression of FAM83H-AS1 in the serum of cancer patients and its underlying mechanisms remains limited. Only a handful of studies have investigated the expression of FAM83H-AS1 in the serum of cancer patients and its clinical significance [22, 24]. For example, Zhou et al. [22] reported that the serum of patients with pancreatic ductal adenocarcinoma exhibited significant upregulation of FAM83H-AS1, which was positively correlated with shorter survival times.

However, the expression levels of FAM83H-AS1 in the serum of OC patients and its associations with prognosis and clinicopathological parameters have not yet been reported. Further investigation is needed to elucidate its potential role in the diagnosis of OC and the assessment of malignancy, thereby providing a theoretical basis for the treatment of OC.

## Materials and methods

### Study participants and specimens

In this study, the recruited cohort consisted of women with suspected gynecological malignancies or suspected OC who visited Jilin Cancer Hospital, Changchun, China. A total of 191 women with pelvic masses confirmed by surgical resection and biopsy histopathology from March 2022 to June 2024 were selected. Women who had undergone gynecological tumor resection or had received radiotherapy or chemotherapy were excluded from the study. Ultimately, 118 patients diagnosed with OC and 73 patients with benign ovarian disease (BOD) were included, along with 61 healthy controls (HCs) who were normal women without gynecological tumors, as determined by imaging and other relevant examinations during the same period. The age, maximum tumor size, menopausal status, cancer location, tumor stage, lymphatic metastasis, CT findings of lymphatic metastasis, distal metastasis, peritoneal metastasis, ascites, and blood biomarkers of the enrolled patients were recorded. Among these, the status of distal metastasis was classified based on preoperative imaging and surgical-pathological findings. Patients were categorized into two groups: a non-distal metastasis group, where tumor spread was confined to the pelvis, and a distal metastasis group, where confirmed metastasis occurred beyond the pelvis. All OC patients underwent regular postoperative follow-up through telephone consultations and electronic medical records until the cutoff date of October 31, 2025. By this date, four patients (3.4%) had been lost to follow-up.

On the day of admission, fasting venous blood was collected from the patients using a vacuum blood collection needle and transferred to sterile tubes. Following blood collection, the samples were allowed to stand at room temperature for one hour. The collected blood was then centrifuged at 4 °C at 3500 rpm for 10 min, and the supernatant was separated. The supernatant was aliquoted and stored at -80 °C prior to detection.

### RNA extraction and RT‑qPCR

Total RNA was extracted using TRIzol LS reagent (Invitrogen, Carlsbad, CA, USA). The extracted RNA was converted into cDNA via the All-in-One First-Strand cDNA Synthesis Kit (#F0202, LABLEAD, Beijing, China). For qPCR, the cDNA was amplified via the Taq SYBR Green^®^ qPCR Premix Kit (#R0202, LABLEAD, Beijing, China). The primers used were as follows: FAM83H-AS1 (F: 5’-GAGATGGACGCCTTCAAG-3’, R: 5’-GGTACTGCTGGTAGA-3’) and the reference gene glyceraldehyde 3-phosphate dehydrogenase (GAPDH) (F: 5’-GGAAGGTGAAGGTCGGAGTC-3’, R: 5’-GTTGAGGTCAATGAAGGGGTC-3’). Relative expression levels of target lncRNAs were calculated via the 2^−ΔΔCt^ method.

### Detection of serum CEA, CA125, and HE4 levels and calculation of the ROMA index

The serum concentrations of CEA, CA125 and HE4 were determined using an AutoLumo A2000 Plus (Antu Bioengineering Company, Zhengzhou, China) fully automated chemiluminescence analyser and the provided diagnostic reagents. All the assays were conducted by qualified technicians in accordance with the manufacturer’s instructions. The ROMA value was calculated from the serum levels of two tumor markers, HE4 and CA125, to assess the risk of OC in women presenting with pelvic masses, as illustrated in the following equation:

$$\begin{aligned}&\mathrm{Premenopausal}(\mathrm{PI})=-12.0+2.38\times\mathrm{LN}(\mathrm{HE}4)+0.0626\times\mathrm{LN}(\mathrm{CA}125),\\& \mathrm{Postmenopausal}(\mathrm{PI})=-8.09+1.04\times\mathrm{LN}(\mathrm{HE}4)+0.732\times\mathrm{LN}(\mathrm{CA}125), \\& \mathrm{LN}=\text{natural log function}, \text{ROMA value}=\mathrm{exp}(\mathrm{PI})/[1+\mathrm{exp}(\mathrm{PI})]\times100\end{aligned}$$

### Statistical analysis

R software (version 4.5.1) and SPSS 24.0 (IBM Corp., Armonk, NY, USA) were utilized for all statistical analyses in this study. FAM83H-AS1 levels across different groups were evaluated via the Kruskal‒Wallis test, whereas the Mann‒Whitney U test was used to examine significant correlations between FAM83H-AS1 levels and clinicopathological parameters. Additionally, receiver operating characteristic (ROC) curve analysis was performed using SPSS 24.0 to calculate the area under the curve (AUC), sensitivity, specificity, and optimal cut-offs (defined by the Youden index). Bootstrap-corrected AUC with 95% confidence intervals (BCa method, 1,000 replicates) was computed in R (version 4.5.1) using the roc and ci.auc functions from the pROC package to assess model robustness. A Z-test was used to compare the AUCs between the two groups. Following univariate logistic regression (IBM SPSS Statistics, version 24), variables with a *P* < 0.1(this liberal threshold was adopted to minimize the risk of omitting potentially important predictors, given the limited sample size) were selected for multivariate analysis. A stepwise multivariable logistic regression was then performed using the forward conditional (likelihood ratio) method for variable selection. Variables that achieved a significance level of *P* < 0.05 in the final model were retained as independent diagnostic factors. To explore the relationships between serum FAM83H-AS1 levels and various indices, Spearman’s correlation test was performed. Furthermore, Student’s t test was used to compare FAM83H-AS1 levels across the two groups. Univariate Cox regression analysis was used to determine the associations between clinicopathological variables and prognosis. Significant variables identified in the univariate Cox regression analysis were then incorporated into multivariate Cox regression analysis to identify independent prognostic factors for patients with OC. Survival differences among different FAM83H-AS1 groups were compared using Kaplan–Meier analysis and the log-rank test. *P* < 0.05 were considered indicative of a significant difference.

## Results

### The serum levels of FAM83H-AS1 were significantly increased in OC patients

**Fig. 1:**
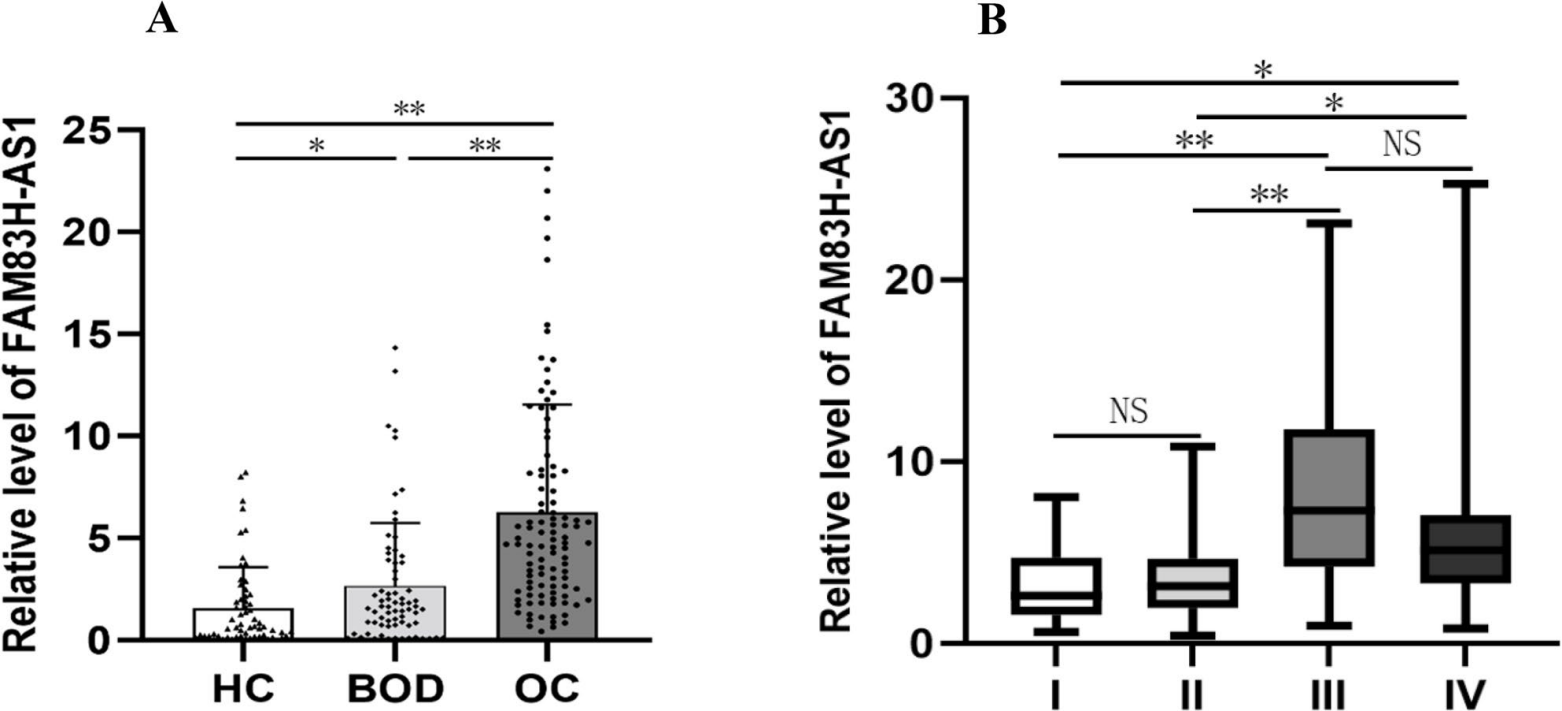
The serum levels of FAM83H-AS1 across various groups are shown. **A** The scatter plot and bar plot depict FAM83H-AS1 expression levels in OC, BOD, and HC. **B** The varying relative levels of FAM83H-AS1 in different TNM stages of OC (stages I, II, III and IV). Statistical significance is indicated as * *P* < 0.05, ** *P* < 0.001

The study revealed that serum FAM83H-AS1 expression levels were markedly higher in OC patients than in BOD patients and HCs (both *P* < 0.05, Fig. 1A). Additionally, serum FAM83H-AS1 levels in BOD patients were significantly elevated compared with those in HCs (*P* < 0.05). Furthermore, the expression levels of FAM83H-AS1 were assessed across different TNM stages and were significantly greater in advanced stages than in early stages (*P* < 0.05, Fig. 1B). Moreover, the ROMA scores were also significantly elevated in OC patients compared with those in BOD patients and HCs (*P* < 0.05, Table 1).

**Table 1:** The serum levels of FAM83H-AS1 and ROMA in the three groups

Group	Number	FAM83H-AS1(ng/ml) (median, range)	ROMA (median, range)
HC	61	0.67(0.19–2.12)	12.15(10.41–15.4)
BOD	73	1.85(0.74–4.41) ^*^	8.63(5.40-14.09)
OC	118	4.92(2.64–9.92) ^*△^	67.32(30.43–95.22) ^*△^

*HC* Healthy controls, *BOD* Benign ovarian disease, *OC* Ovarian cancer, *ROMA* Risk of ovarian malignancy algorithm. ^*^P < 0.05 compared with HC, ^△^*P* < 0.05 compared with patients with BOD

### Correlations between serum FAM83H-AS1 levels and clinicopathological parameters of OC patients

The associations between serum FAM83H-AS1 levels and the clinicopathological parameters of OC patients are summarized in Table 2. The serum expression levels of FAM83H-AS1 did not significantly correlate with age, maximum tumor size, menopausal status, CT-reported lymphatic metastasis, or ascites (all *P* > 0.05). However, the serum expression levels of FAM83H-AS1 were significantly correlated with cancer location, TNM stage, lymphatic metastasis, distal metastasis, and peritoneal metastasis in OC patients (all *P* < 0.05, Table 2).

**Table 2:** Correlation of serum FAM83H-AS1 levels with clinicopathological parameters of OC patients

Clinipathological parameters	Case number	FAM83H-AS1 level [median(IQR)]	*P* value
Age(year)			
< 55	49	5.28(2.85,11.12)	0.642
≥ 55	69	5.58(2.59,13.78)	
Maximum tumor size(cm)			
< 5	37	4.12(1.90,8.82)	0.369
≥ 5	81	5.28(2.84,11.12)	
Menopausal status			
Yes	92	5.58(2.69,11.71)	0.625
No	26	4.56(2.66,11.76)	
Cancer location			
Unilateral	39	4.63(2.20,7.31)	**0.012**
Bilateral	79	5.86(3.16,15.14)	
TNM stage			
I-II	33	3.04(1.81,4.69)	**< 0.001**
III-IV	85	7.31(4.13,17.05)	
Lymphatic metastasis			
Yes	72	7.36(4.34,19.43)	**< 0.001**
No	46	3.36(1.89,6.09)	
CT report lymphatic metastasis			
Yes	23	5.78(3.56,20.68)	0.307
No	95	5.28(2.64,11.39)	
Distal metastasis			
Yes	78	5.96(3.54,18.90)	**0.001**
No	40	3.94(2.20,6.28)	
Peritoneal metastasis			
Yes	74	5.96(3.46,16.25)	**0.006**
No	44	4.63(2.20,6.68)	
Ascites			
Yes	58	5.78(3.08,8.93)	0.261
No	60	5.02(2.55,6.68)	

Data in bold indicate statistically significant values

*CT* Computed tomography, *IQR* Interquartile range

## FAM83H-AS1 may serve as a potential biomarker for OC screening

**Fig. 2:**
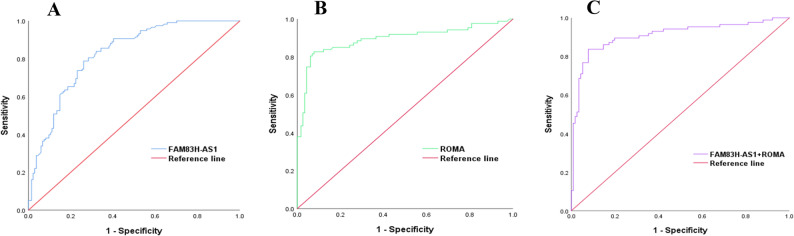
Diagnostic performance of FAM83H-AS1 and ROMA for discriminating OC from BOD and HCs. **A** Serum FAM83H-AS1. **B** Serum ROMA. **C** Combination of FAM83H-AS1 and ROMA

The diagnostic value of FAM83H-AS1 for OC was assessed using ROC curves. FAM83H-AS1 alone effectively distinguished OC patients from those with BOD and HCs, as illustrated in Fig. 2 and detailed in Table 3. Its combination with ROMA further enhanced diagnostic performance, achieving an optimal AUC of 0.909, with a sensitivity of 83.72% and a specificity of 92.24% for OC screening. Internal validation using 1,000 bootstrap replicates confirmed the stability of these results. The bootstrap AUCs (FAM83H-AS1: 0.831; ROMA: 0.891; combined: 0.909) were highly consistent with the original values, thereby affirming the reliability of our models.

**Table 3:** Diagnostic performance of FAM83H-AS1, ROMA and FAM83H-AS1 + ROMA in serum for OC versus BOD and HCs

Marker	AUC (95%CI)	Bootstrap AUC (95%CI)	Sensitivity (η/%)	Specificity (η/%)	cut-off value	*P* value
FAM83H-AS1	0.824(0.774–0.875)	0.831(0.775–0.875)	78.81	73.88	2.50	**< 0.001**
ROMA	0.894(0.843–0.944)	0.891(0.835–0.939)	82.76	92.17	20.46	**< 0.001**
FAM83H-AS1 + ROMA	0.909(0.863–0.955)	0.909(0.853–0.946)	83.72	92.24	0.31	**< 0.001**

Data in bold indicate statistically significant values

*ROMA* Risk of ovarian malignancy algorithm, *AUC* Area under the curve, *95%CI* 95% Confidence Interval

### Subgroup analysis: diagnostic performance of FAM83H-AS1 in OC versus benign gynecological diseases, and in early-stage OC versus BOD and HCs

**Fig. 3:**
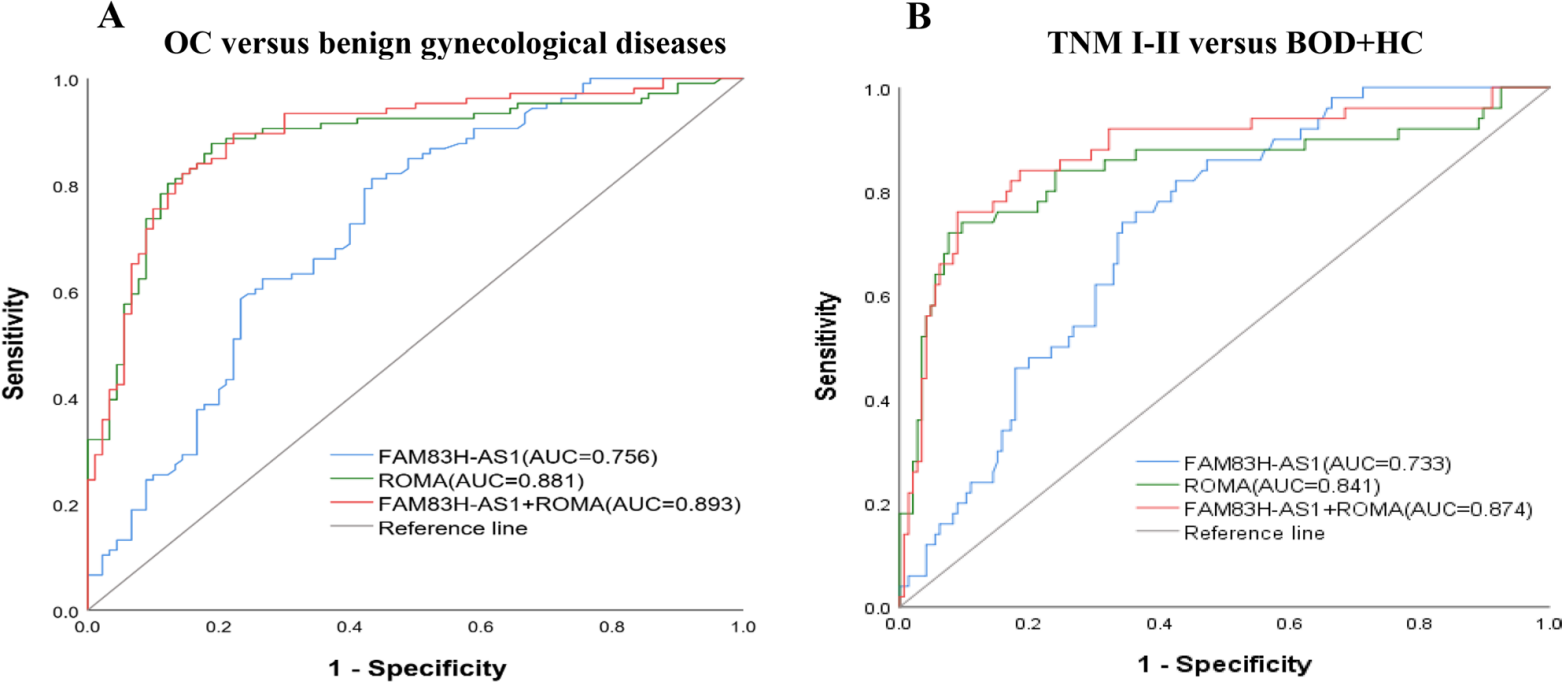
Subgroup ROC curve analyses for FAM83H-AS1 and ROMA. **A** Diagnostic performance of serum FAM83H-AS1, ROMA, and their combination in discriminating OC from benign gynecological diseases. **B** Diagnostic performance of the same biomarkers in discriminating TNM I-II from a combined group of BOD and HCs

To clarify the diagnostic specificity of FAM83H-AS1 and its performance in benign gynecological diseases and early-stage, we conducted additional subgroup analyses. The newly added 32 patients with benign gynecological diseases were pooled with the original 73 BOD cohort, included benign gynecological diseases comprised endometriosis (27 cases), mature cystic teratoma (17 cases), uterine leiomyoma (15 cases), serous cystadenoma (13 cases), mucinous cystadenoma (8 cases), endometrial polyp (6 cases), cervical intraepithelial neoplasia (4 cases), ovarian fibroma (3 cases), and others (12 cases). An additional subgroup ROC curve analysis was performed comparing the combined benign gynecological diseases group against the original OC group, with internal validation through bootstrap resampling analysis (1000 repetitions). As shown in Fig. 3; Table 4, when specifically comparing OC versus benign gynecological diseases, FAM83H-AS1 maintained robust diagnostic performance with an AUC of 0.756, 95% CI: (0.694–0.818), which was further validated by bootstrap analysis (AUC = 0.756, 95% CI: 0.687–0.817). The combination of FAM83H-AS1 with ROMA achieved the highest diagnostic accuracy in this challenging comparison (AUC = 0.893, 95% CI: 0.846–0.939).

**Table 4:** Diagnostic performance of FAM83H-AS1, ROMA and FAM83H-AS1 + ROMA in serum for OC versus benign gynecological diseases

Marker	AUC (95%CI)	Bootstrap AUC (95%CI)	Sensitivity(η/%)	Specificity(η/%)	cut-off value	*P* value
FAM83H-AS1	0.756(0.694-0.818)	0.756(0.687-0.817)	80.29	60.58	2.47	**< 0.001**
ROMA	0.881(0.830-0.931)	0.880(0.828-0.923)	87.74	81.11	14.20	**< 0.001**
FAM83H-AS1+ROMA	0.893(0.846-0.939)	0.893(0.843-0.933)	82.08	85.60	0.68	**< 0.001**

Data in bold indicate statistically significant values

*ROMA* Risk of ovarian malignancy algorithm, *AUC* Area under the curve, *95%CI* 95% Confidence Interval

Furthermore, we combined 18 newly recruited early-stage (TNM I-II) patients with the original cohort of 34 early-stage patients, thereby forming a supplemented early-stage cohort. The diagnostic performance of the biomarkers was specifically evaluated to distinguish this early-stage OC cohort from a combined control group consisting of BOD and HCs, with internal validation conducted via bootstrap resampling (1000 repetitions). As illustrated in Fig. 3; Table 5, FAM83H-AS1 exhibited significant diagnostic capability for early-stage detection, achieving an AUC of 0.733 (95% CI: 0.663–0.802). This finding was further corroborated by bootstrap analysis, which yielded an AUC of 0.734 (95% CI: 0.655–0.794). Moreover, the combination of FAM83H-AS1 with ROMA resulted in an even higher diagnostic accuracy, with an AUC of 0.909 (95% CI: 0.863–0.955).

**Table 5:** Diagnostic performance of FAM83H-AS1, ROMA and FAM83H-AS1 + ROMA in serum for TNM I-II versus BOD and HCs

Marker	AUC (95%CI)	Bootstrap AUC (95%CI)	Sensitivity (η/%)	Specificity (η/%)	cut-off value	*P* value
FAM83H-AS1	0.733(0.663–0.802)	0.734(0.655–0.794)	84.62	55.76	1.77	**< 0.001**
ROMA	0.841(0.764–0.919)	0.844(0.736–0.904)	72.00	92.47	22.00	**< 0.001**
FAM83H-AS1 + ROMA	0.874(0.810–0.938)	0.875(0.803–0.926)	76.00	91.10	0.22	**< 0.001**

Data in bold indicate statistically significant values

*ROMA* Risk of ovarian malignancy algorithm, *AUC* Area under the curve, *95%CI* 95% Confidence Interval

### Stepwise multivariate analysis for OC diagnosis

To further evaluate the independent contribution of FAM83H-AS1 within the combined diagnostic model, we performed univariate and multivariate regression analyses. Univariate analysis revealed that FAM83H-AS1, age and ROMA were all significantly associated with OC diagnosis (all *P* < 0.001). Subsequently, the stepwise multivariate analysis incorporated FAM83H-AS1 and ROMA into the final diagnostic model (both *P* < 0.01). Results of the stepwise multivariate analysis are presented in Table 6. Each unit increase in FAM83H-AS1 was associated with a 6.9% increase in the odds of having OC (OR = 1.069, 95% CI: 1.006–1.135, *P* = 0.030). Similarly, each unit increase in ROMA was associated with a 7.0% increase in the odds of OC (OR = 1.070, 95% CI: 1.047–1.092, *P* < 0.001).

**Table 6:** Stepwise multivariate analysis of biomarkers for distinguishing OC from those with BOD and HCs

Characteristics	B	S.E.	Wald χ²	*P* value	OR	95%CI
FAM83H-AS1	0.067	0.031	4.714	**0.030**	1.069	1.006–1.135
ROMA	0.067	0.011	39.133	**< 0.001**	1.070	1.047–1.092

Data in bold indicate statistically significant values

*ROMA* Risk of ovarian malignancy algorithm, *B* Beta coefficient, *S.E.* Standard Error, *Wald χ²* Wald Chi-Square statistic, *OR* Odds Ratio, *95%CI* 95% Confidence Interval

### Serum FAM83H-AS1 levels were correlated with indicators of disease severity in OC

**Fig. 4:**
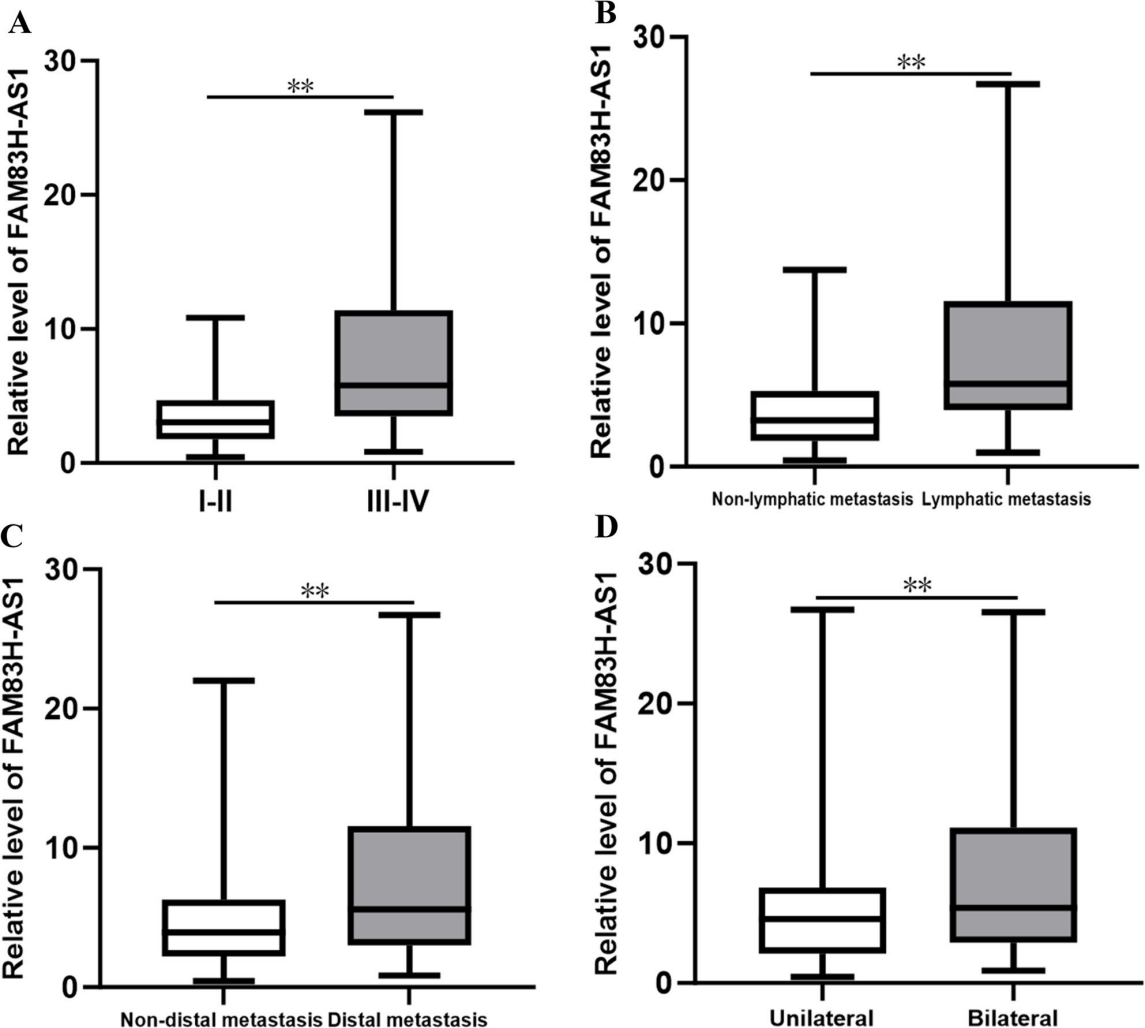
Serum FAM83H-AS1 levels across different participant groups in patients with OC. **A** TNM III-IV versus TNM I-II. **B** lymphatic metastasis versus non-lymphatic metastasis. **C** distal metastasis versus non-distal metastasis status in OC patients. **D** bilaterality versus unilaterality. ** *P* < 0.001

**Fig. 5:**
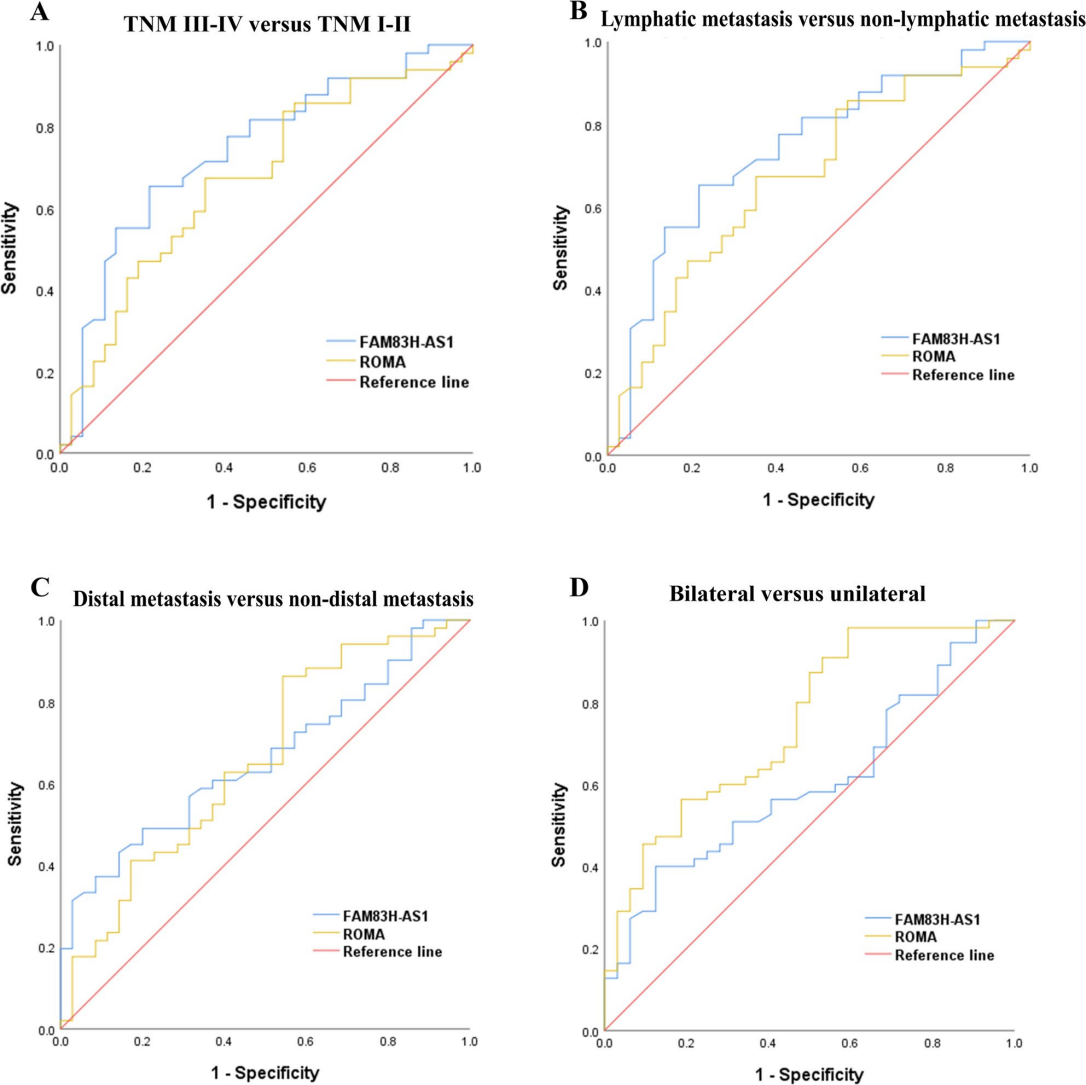
ROC curve analyses for serum FAM83H-AS1 levels in different groups of OC patients. **A** TNM III-IV versus TNM I-II. **B** lymphatic metastasis versus non-lymphatic metastasis. **C** distal metastasis versus non-distal metastasis. **D** bilateral versus unilateral

Given the significant elevation of serum FAM83H-AS1 in OC, this study further examined the correlation between serum FAM83H-AS1 expression and various clinicopathological features associated with OC progression, including TNM stage, lymphatic metastasis, cancer location, and distant metastasis. Additionally, ROC curve analysis was employed to assess the ability of FAM83H-AS1 to predict lymphatic metastasis, distal metastasis, TNM stage, and cancer location (see Figs. 4 and 5; Table 7). FAM83H-AS1 levels were significantly elevated in patients exhibiting more advanced and aggressive disease characteristics. Specifically, FAM83H-AS1 expression level was markedly higher in patients with advanced TNM stage (III-IV) (median [IQR]: 7.31 [4.13–17.05] ng/ml) compared to those with early-stage disease (I-II) (3.04 [1.81–4.69] ng/ml; *P* < 0.001). Similarly, the levels were significantly increased in patients presenting with lymphatic metastasis (7.36 [4.34–19.43] ng/ml) versus those without (3.36 [1.89–6.09] ng/ml; *P* < 0.001). FAM83H-AS1 expression was significantly higher in the distal metastasis (5.96 [3.54–18.90] ng/ml) than in the non-distal metastasis group (3.94 [2.20–6.28] ng/ml; *P* = 0.001). Furthermore, patients with bilateral tumors exhibited higher levels (5.86 [3.16–15.14] ng/ml) than those with unilateral involvement (4.63 [2.20–7.31] ng/ml; *P* = 0.012) (Fig. 4). The ROC curve for serum FAM83H-AS1 demonstrated a significant distinction between TNM III-IV and TNM I-II, and the AUC was 0.800. The AUCs of serum FAM83H-AS1 for diagnosing lymphatic metastasis and distal metastasis were 0.741 and 0.693, respectively (all *P* < 0.05, Fig. 5; Table 7). Notably, the ROC curve of serum FAM83H-AS1 exhibited superior performance in distinguishing TNM III-IV from TNM I-II, as well as discriminating between lymphatic metastasis and non-lymphatic metastasis (Fig. 5A, B). However, the ROC curve analysis of serum FAM83H-AS1 did not significantly differ between bilateral and unilateral cases, yielding an AUC of 0.604 (*P* > 0.05, see Fig. 5; Table 7).

**Table 7:** Relationships between FAM83H-AS1 and TNM stage, lymphatic metastasis, distal metastasis and cancer location and its diagnostic value

Marker	AUC (95%CI)	Sensitivity (η/%)	Specificity (η/%)	cut-off value	*P* value
TNM III + IV versus I + II	0.800(0.720–0.879)	69.41	81.82	4.84	**< 0.001**
Lymphatic metastasis versus non-lymphatic metastasis	0.741(0.634–0.848)	66.67	73.33	5.39	**< 0.001**
Distal metastasis versus non-distal metastasis	0.693(0.597–0.788)	37.18	94.87	11.43	**0.001**
Unilateral versus bilateral	0.604(0.484–0.723)	40.00	87.50	8.23	0.107

Data in bold indicate statistically significant values

*AUC* Area under the curve, *95%CI* 95% Confidence Interval

### Correlations between serum FAM83H-AS1 levels and various indices in OC patients

Table 8 shows a significant positive correlation between serum FAM83H-AS1 expression and various clinical factors, including TNM stage, cancer location, lymphatic metastasis, distal metastasis, peritoneal metastasis, CA125, HE4, and ROMA (all *P* < 0.05). Nevertheless, no substantial relationships were found between the serum FAM83H-AS1 level and maximum tumor size, menopausal status, or CEA level (all *P* > 0.05).

**Table 8:** Associations between serum FAM83H-AS1 levels and various indices in OC patients

Indices	FAM83H-AS1	
	*r*	*P* value
TNM stage	0.470	**< 0.001**
Maximum tumor size(cm)	0.117	0.133
Menopausal status	0.121	0.100
Cancer location	0.193	**0.029**
Lymphatic metastasis	0.411	**< 0.001**
Distal metastasis	0.343	**< 0.001**
Peritoneal metastasis	0.291	**< 0.001**
CEA(ng/ml)	0.097	0.258
CA125(U/ml)	0.254	**0.001**
HE4(pmol/L)	0.280	**0.001**
ROMA	0.361	**< 0.001**

Data in bold indicate statistically significant values

*CEA* Carcinoembryonic antigen, *CA125* Cancer antigen 125, *HE4* Human epididymis protein 4, *ROMA* Risk of ovarian malignancy algorithm

### Compared with preoperative levels, serum FAM83H-AS1 expression levels in OC patients were significantly lower after surgery

**Fig. 6:**
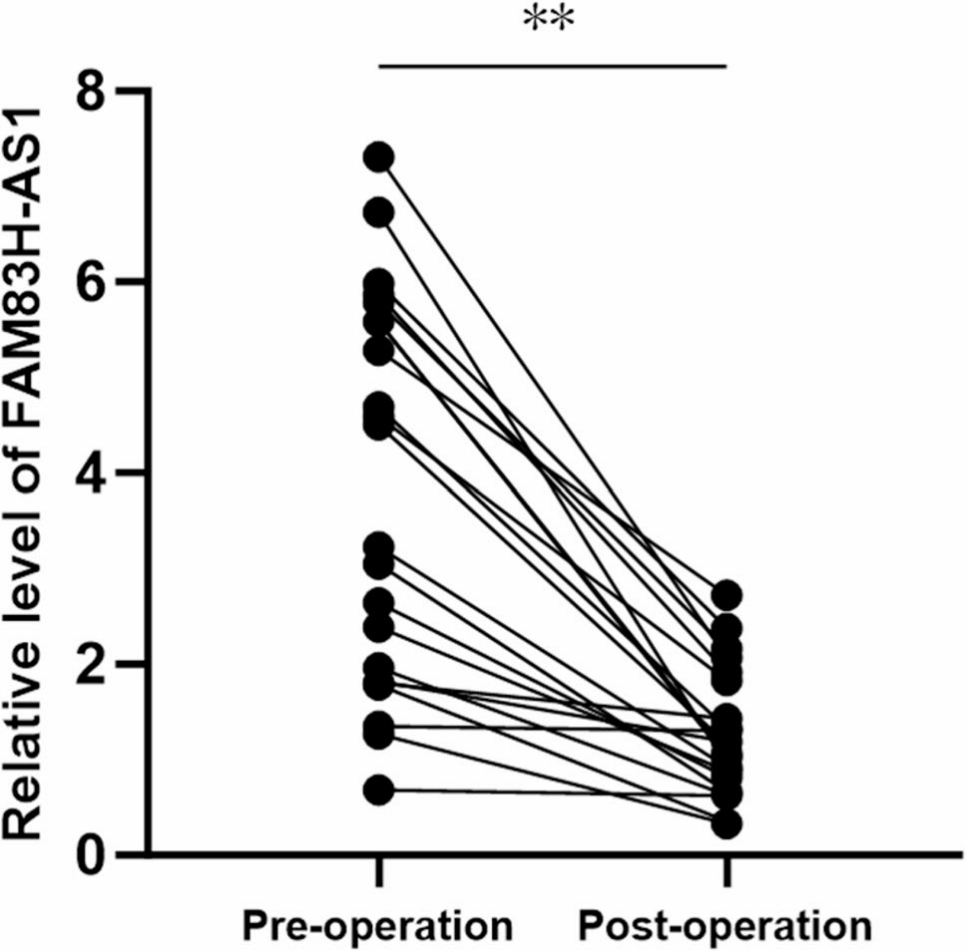
Changes in serum FAM83H-AS1 levels in preoperative and postoperative OC patients. The expression levels of serum FAM83H-AS1 were assessed through RT-qPCR in preoperative and postoperative patients. The data from the two groups were analysed via Student’s *t* test. ** *P* < 0.001

To evaluate the impact of surgery on serum FAM83H-AS1 expression, we randomly selected 22 matched pairs of preoperative and postoperative samples from the enrolled OC patients. The results of the correlation analysis indicated that the serum levels of FAM83H-AS1 in postoperative patients were significantly lower than those in preoperative patients (Fig. 6). These findings clarify that circulating FAM83H-AS1 originates from tumor tissues, suggesting its potential as an indicator for the early diagnosis of OC.

### Prognostic value of serum FAM83H-AS1 in OC

Univariate Cox regression analysis revealed that high serum levels of FAM83H-AS1, lymph node metastasis, advanced TNM stage, older age, bilateral lesions, distant metastasis, peritoneal metastasis, and ascites were associated with shorter progression-free survival (PFS) in OC patients (all *P* < 0.1). To further identify independent prognostic factors, the variables with statistical significance in the univariate analysis were included in the subsequent multivariate Cox regression model. After adjusting for other confounding factors, only high serum levels of FAM83H-AS1 (HR = 8.251, 95% CI: 1.085–62.762; *P* = 0.041), advanced TNM stage (HR = 2.285, 95% CI: 1.405–3.718; *P* = 0.001), and the presence of ascites (HR = 2.119, 95% CI: 1.022–4.397; *P* = 0.044) emerged as independent risk factors for poorer PFS (Table 9).

**Table 9:** Univariate and multivariate Cox regression analyses for PFS in OC patients

Clinipathological parameters	Univariate Analysis		Multivariate Analysis	
	HR (95% CI)	*P* value	HR (95% CI)	*P* value
FAM83H-AS1				
Low	Reference		Reference	
High	13.334 (1.818–97.806)	**0.011**	8.251 (1.085–62.762)	**0.041**
Lymphatic Metastasis				
No	Reference		Reference	
Yes	4.667 (1.809–12.046)	**0.001**	1.522(0.541–4.286)	0.426
TNM Stage	2.921(1.763–4.841)	**< 0.001**	2.285(1.405–3.718)	**0.001**
Age (years)	1.038 (1.003–1.074)	**0.032**	1.013(0.972–1.057)	0.535
Tumor Size (cm)	0.990 (0.908–1.079)	0.817		
Menopause age (years)	0.957 (0.864–1.059)	0.391		
ROMA	1.011 (0.998–1.025)	0.266		
Menopausal status				
No	Reference			
Yes	1.658 (0.689–3.989)	0.259		
Cancer location				
Unilateral	Reference		Reference	
Bilateral	3.162 (1.226–8.160)	**0.017**	1.472 (0.471–4.604)	0.506
Distal metastasis				
No	Reference		Reference	
Yes	4.406 (1.554–12.487)	**0.005**	1.126 (0.318–3.989)	0.855
Peritoneal metastasis				
No	Reference		Reference	
Yes	4.366 (1.696–11.240)	**0.002**	1.313 (0.407–4.230)	0.649
Ascites				
No	Reference		Reference	
Yes	2.147 (1.083–4.253)	**0.020**	2.119(1.022–4.397)	**0.044**

Data in bold indicate statistically significant values

*ROMA* Risk of ovarian malignancy algorithm, *HR* Hazard Ratio, *95%CI* 95% Confidence Interval, *Reference* The baseline group for HR calculation

Furthermore, univariate Cox regression analysis showed that high serum levels of FAM83H-AS1, lymph node metastasis, advanced TNM stage, older age, and higher menopause age were associated with poorer overall survival (OS) in OC patients (all *P* < 0.1). After adjusting for other confounding factors, only advanced TNM stage (HR = 5.377, 95% CI: 1.303–22.196; *P* = 0.020) was independently associated with OS (Table 10).

**Table 10:** Univariate and multivariate Cox regression analyses for OS in OC patients

Clinipathological parameters	Univariate Analysis		Multivariate Analysis	
	HR (95% CI)	*P* value	HR (95% CI)	*P* value
FAM83H-AS1				
Low	Reference		Reference	
High	1.020 (1.002–1.039)	**0.031**	1.154 (0.901–8.890)	0.967
Lymphatic Metastasis				
No	Reference		Reference	
Yes	5.764 (0.728–45.659)	**0.097**	1.299(0.156–10.857)	0.809
TNM Stage	5.889(1.425–24.420)	**0.014**	5.377(1.303–22.196)	**0.020**
Age (years)	1.122 (1.042–1.207)	**0.002**	1.063(0.974–1.160)	0.170
Tumor Size (cm)	0.946 (0.797–1.123)	0.524		
Menopause age (years)	0.965(0.809–1.150)	0.687		
ROMA	0.991 (0.968–1.014)	0.435		
Menopausal status				
No	Reference			
Yes	0.033 (0.001–27.923)	**0.072**	0.451(0.064–2.160)	0.972
Cancer location				
Unilateral	Reference			
Bilateral	0.026 (0.001–7.207)	0.203		
Distal metastasis				
No	Reference			
Yes	42.777 (0.194-9435.91)	0.173		
Peritoneal metastasis				
No	Reference			
Yes	4.454 (0.560-35.434)	0.158		
Ascites				
No	Reference			
Yes	1.761 (0.513–6.043)	0.368		

Data in bold indicate statistically significant values

*ROMA* Risk of ovarian malignancy algorithm, *HR* Hazard Ratio, *95%CI* 95% Confidence Interval, *Reference* The baseline group for HR calculation

**Fig. 7:**
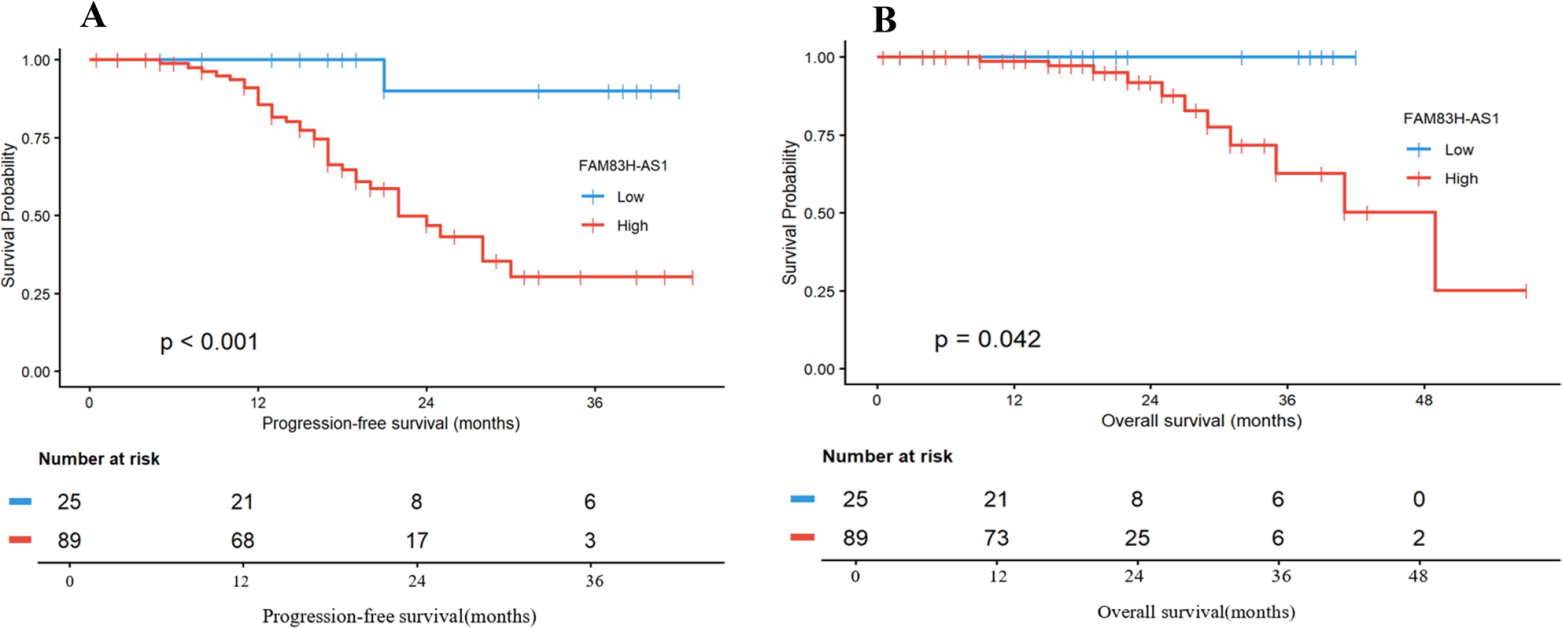
Kaplan-Meier analysis showed that high FAM83H-AS1 expression was associated with significantly worse **A** progression-free survival (PFS; Log-rank *P* < 0.001) and **B** overall survival (OS; Log-rank *P* < 0.05) in OC patients

To evaluate the impact of FAM83H-AS1 expression levels on PFS and OS in OC patients, we conducted Kaplan-Meier analyses. Patients were categorized into high and low expression groups based on FAM83H-AS1. The Log-rank test demonstrated that the differences in both PFS and OS between the two groups were statistically significant (PFS: *P* < 0.001) and OS: *P* < 0.05) (Fig. 7).

## Discussion

Multi-omics technologies and computational tools have become key cutting-edge technologies for understanding the complex molecular mechanisms of cancer. As a critical component of multi-omics, liquid biopsy and biomarker research are at the forefront of this field. Notably, driven by transcriptomic technologies, lncRNAs have been extensively explored across multiple fields, including tumor mechanisms, drug safety evaluation, and biomarker discovery [25, 26]. Despite the limited characterization of functional lncRNAs to date, recent research underscores their potential as innovative diagnostic agents owing to their stability in biological fluids and their involvement in tumor development. Numerous studies highlight the critical roles of various lncRNAs in mediating OC progression through their impact on essential signalling pathways, including the PI3K/AKT, EMT-associated, and β-catenin signalling pathway. Collectively, these findings position lncRNAs as transformative candidates for cancer diagnostics and therapeutics [27–30]. Among them, FAM83H-AS1 has been strongly linked to tumor advancement and poor outcomes in various cancers, including lung cancer [31], hepatocellular carcinoma [32], gastric cancer [33], bladder cancer [17], and breast cancer [24]. However, reports concerning FAM83H-AS1 in OC remain scarce. At present, there is evidence indicating that the expression of FAM83H-AS1 in OC tissues is regulated at a higher level than that in normal tissues, with even higher expression levels observed in advanced-stage OC. The increase in the expression of FAM83H-AS1 is related to the progression of the disease and the unfavorable clinical outcomes of cancer patients. A study by Gong et al. [20] reported significant overexpression of FAM83H-AS1 in both OC tissues and cell lines, with this upregulation significantly associated with critical clinicopathological parameters, including tumor grade, FIGO stage, and distant metastasis. Patients with high FAM83H-AS1 expression presented significantly shorter overall survival. Most studies on FAM83H-AS1 expression have focused primarily on OC tissues and cell lines; however, its role in the serum of OC patients remained unexplored prior to this investigation.

Here, for the first time, we demonstrate that FAM83H-AS1 may serve as a potential and noninvasive diagnostic biomarker for OC. The results demonstrated that the serum levels of FAM83H-AS1 were significantly elevated in OC patients compared with those in BOD patients and HCs, with further increases observed in advanced TNM stages and metastatic cases. A study by Zhou et al. [22] similarly reported that serum levels of FAM83H-AS1 were significantly elevated in patients with pancreatic ductal adenocarcinoma, indicating a strong association with shorter survival. Importantly, despite the limited research on FAM83H-AS1 in OC, other studies have shown that FAM83H-AS1 expression levels are increased in OC tissues and cell lines [9]. Interestingly, serum FAM83H-AS1 levels in OC patients significantly decreased after surgery. These findings support the possible tumor tissue origin of FAM83H-AS1 and increase its practicality as a dynamic monitoring tool. These findings are consistent with other studies suggesting that FAM83H-AS1 may increase FAM83H expression by stabilizing its mRNA, thereby reducing the ubiquitylation of β-catenin regulated by FAM83. These coordinated molecular interactions amplify the FAM83H-AS1/FAM83H/β-catenin signalling cascade, ultimately driving the proliferation, invasion, and metastasis of cancer cells [22]. Additionally, silencing FAM83H-AS1 can stabilize the HuR protein, thereby inhibiting the proliferation and metastasis of OC cells [34].

Furthermore, we evaluated the diagnostic ability of serum FAM83H-AS1 and ROMA by calculating their AUCs. These findings suggest that FAM83H-AS1 has strong diagnostic performance in differentiating OC patients from those with BOD and HCs. The combined test of FAM83H-AS1 and ROMA exhibited increased diagnostic efficiency and demonstrated an excellent AUC with high sensitivity and specificity for OC screening. Bootstrap resampling for internal validation confirmed the high stability of the AUC values across all models. These AUCs were associated with narrow confidence intervals significantly above 0.5, indicate that the findings are robust and unlikely to be due to chance. Moreover, the stepwise multivariate analysis highlighted the value of FAM83H-AS1 as an independent contributor to OC diagnosis. The robustness of ROMA, a clinically commonly used screening and auxiliary diagnostic indicator for OC, was expected, while the independence of FAM83H-AS1 consolidated its potential role in multi-marker panels. This also indirectly corroborated that the superior performance of the combined test of FAM83H-AS1 and ROMA likely stems from this complementary relationship.

In the subgroup analysis, we specifically investigated the ability of FAM83H-AS1 to distinguish OC patients from those with benign gynecological diseases. The results showed that FAM83H-AS1 maintained robust diagnostic performance. FAM83H-AS1 may be elevated in conditions involving rapid cell proliferation or inflammation, not being limited to malignancies. Future research must include a more diverse and larger sample size of patients with benign gynecological diseases. Furthermore, FAM83H-AS1 retained significant diagnostic value in distinguishing early-stage OC patients from the combined group of BOD and HCs. Notably, the diagnostic efficacy (AUC) of FAM83H-AS1 was slightly inferior to that of the ROMA index. This observation may be attributed to the limited sample size in this study, which could have introduced bias.

Our study is the first to reveal that serum FAM83H-AS1 is an independent risk factor for PFS in patients with OC. This finding carries significant clinical implications, suggesting that tumors with high FAM83H-AS1 expression are more aggressive and prone to recurrence after initial treatment. In contrast, the non-independent effect on OS may be related to the relatively short follow-up duration and the fact that patients have not yet undergone multiline therapy. Furthermore, FAM83H-AS1 was associated with TNM stage, lymphatic metastasis, distal metastasis and peritoneal metastasis, indicating its role in predicting cancer malignancy and dynamic progression. Our current findings in the serum compartment align with prior studies on OC tumor tissues. Previous reports have shown that high FAM83H-AS1 expression in tissues is correlated with distant metastasis, FIGO stage, and pathological grade [20]. Mechanistically, FAM83H-AS1 overexpression in OC tissues is known to modulate the infiltration of immune cells—such as dendritic cells, neutrophils, and macrophages—through gene regulatory networks, thereby promoting tumor progression [9]. Consequently, the elevated expression of FAM83H-AS1 in OC tissues is closely linked to poor patient prognosis [9].

The results from this study suggest that serum levels of FAM83H-AS1 increase with the progression of OC malignancy. To validate this, we conducted a correlation analysis of these indicators, which revealed that high expression of serum FAM83H-AS1 was significantly positively correlated with TNM stage, lymphatic metastasis, distant metastasis, and peritoneal metastasis. Notably, FAM83H-AS1 expression was moderately correlated with TNM stage, lymphatic metastasis, and distal metastasis. Interestingly, the expression level of FAM83H-AS1 also showed a moderate correlation with ROMA, a clinically validated algorithm for assessing OC risk [35]. This correlation may suggest a potential interplay between FAM83H-AS1 and the ROMA components, possibly involving shared oncogenic pathways during OC development. Consequently, FAM83H-AS1 might serve a complementary role to ROMA as an effective indicator for evaluating OC risk in patients with pelvic masses. Additionally, serum FAM83H-AS1 exhibits high diagnostic efficacy in relation to tumor TNM stage and lymphatic metastasis. These findings support the potential of serum FAM83H-AS1 as a promising biomarker for assessing the malignancy of OC.

This study presents several limitations. Firstly, this is a single-center study, and the stability of the ROC curve results lacks validation in an external independent cohort. Secondly, the limited sample size, particularly evident in the early-stage patient subgroup, may affect the statistical power and generalizability of the research findings. Thirdly, the prognostic value of FAM83H-AS1 for OS was not independent of established clinicopathological factors, a finding that may be influenced by the relatively short follow-up period. Therefore, its long-term prognostic significance requires further investigation.

In conclusion, our study is the first to identify serum FAM83H-AS1 may serves as a novel noninvasive biomarker for the early detection of OC. Additionally, our findings highlight the potential of serum FAM83H-AS1 as a marker for monitoring OC progression and as a therapeutic target. Future research should focus on exploring the precise functions and mechanisms of serum FAM83H-AS1 in OC. By positioning serum FAM83H-AS1 as a central hub rather than a standalone biomarker, its close association with tumor stage and metastasis underscores its potential in addressing tumor heterogeneity. The integration of proteomic data and advanced tools such as machine learning will be crucial for elucidating the molecular drivers of malignant progression and enhancing its applicability in precision oncology.

## Supplementary Information

Supplementary Material 1.

Supplementary Material 2.

## Data Availability

The datasets used and/or analysed during the current study are available from the corresponding author on reasonable request.
