# Supplementary material for: Serum long noncoding RNA FAM83H-AS1 serves as a potential noninvasive diagnostic biomarker for ovarian cancer
Source: J Ovarian Res. 2026 Feb 3;19:83. doi: 10.1186/s13048-026-01995-1 (PMC12955189; doi:10.1186/s13048-026-01995-1)
Supplement: Supplementary file 1 — Supplementary Material 1. [file 13048_2026_1995_MOESM1_ESM.doc]

**Jilin Province Cancer Hospital Institutional Review Board**

**Ethics Committee Inspection**

Approval No.: 202208-012-01 (Research Paper）

| Research Title | Expression and Clinical Significance of FAM83H, IL-33, and ST2 in Peripheral Blood of Ovarian Cancer Patients | | |
| --- | --- | --- | --- |
| Department | Laboratory Department | | |
| Principle Investigator | Yu Xiuyan | | |
| Institution | Jilin Cancer Hospital | | |
| Inspection type | Research Paper | Review type | quick review |
| Inspection Date | August 9, 2022 | | |
| Inspection Files | Research Paper program and Informed Consent Waiver Applications, etc. | | |
| Review comments:  Accordance with the Law of the People's Republic of China on Medical Practitioners, the Regulations on the Administration of Medical Institutions, the Quality Management Standards for Drug Clinical Trials, the Ethical Review Measures for Biomedical Research Involving Human Beings, the Guiding Principles for the Ethical Review of Drug Clinical Trials, the Declaration of the World Medical Association, and the Operational Guidelines for the Ethics Committee for Biomedical Research Review of the World Health Organization The Ethics Committee agreed to carry out this research in accordance with the approved documents. | | | |
| Please follow the GCP principles and follow the protocol approved by the Ethics Committee to conduct clinical research and protect the health and rights of the subjects.  1. After approval, submit an ethics review application for study initiation.  2. Any amendments to the principal investigator, study protocol, informed consent forms, recruitment materials, etc., must be reported via an amendment review application.  3. Submit a SUSAR report for any suspected unexpected serious adverse reactions.  4. Report protocol deviations that may impact subjects' rights/health or scientific validity.  5. Submit a suspension/termination report if the study is paused or terminated prematurely.  6. Submit a final report upon study completion. | | | |
| Chairperson's Signature | Li Hui | | |
| Committee Chairman (signature) |  | date |  |

Address：No.1066 Jinhu Road, Changchun,

Tel：0431-80596067

postcode：130000

**List of Attending Committee Members of Jilin Provincial Cancer Hospital Ethics Committee**

**1.Declaration of Committee Members of Jilin Provincial Cancer Hospital Ethics Committee:**

The duties, composition, procedures, and records of the Jilin Province Cancer Hospital IRB comply with ICH-GCP, Chinese GCP, and national regulations. Committee members shall maintain confidentiality of all reviewed materials and meeting contents.

**2. Review Time and Location:** August 8, 2022 (Tencent Online Meeting, ID: 794-760-4284F)

**3.List of IRB Members and Attendance:**

| Name | Gender | Affiliation and Title | Committee Role | Attendance |
| --- | --- | --- | --- | --- |
| Li Hui | Female | Researcher, Jilin Cancer Hospital | Chairman | Present |
| Liu Yanling | Female | Chief Physician, Jilin Cancer Hospital | Vice Chairman | Present |
| Li Guangwei | Female | Discipline Inspector, Jilin Cancer Hospital | Member | Present |
| Jing Niancai | Male | Chief Physician, Jilin Cancer Hospital | Member | Present |
| Sun Baosheng | Male | Chief Physician, Jilin Cancer Hospital | Member | Present |
| Zhang Caixia | Female | Chief Physician, Jilin Cancer Hospital | Member | Present |
| Shen Zhigang | Male | Chief Pharmacist, Jilin Cancer Hospital | Member | Present |
| Xing Juying | Female | Deputy Chief Nurse, Jilin Cancer Hospital | Member | Present |
| Zhang Shuang | Female | Deputy Chief Physician, Jilin Cancer Hospital | Member | Present |
| Li Shuang | Female | Community Representative | Member | Present |
| Zhu Zhen | Male | Professor, Jilin University Law School | Member | Present |

**Appendix:**

**《Expression and Clinical Significance of FAM83H, IL-33, and ST2 in Peripheral Blood of Ovarian Cancer Patients**》

| **number** | **File name** | **Version and date** |
| --- | --- | --- |
| 1 | Jilin Province Cancer Hospital published academic papers ethics filing application form | NA |
| 2 | Informed consent waiver application | NA |
| 3 | Clinical Research Approval Form | NA |
| 4 | Health Technology Project Application | Application Date: August 9, 2022 |
| 5 | Research program | NA |
| 6 | Informed Consent Form | Version: 20220808, Date: 2022.8.8 |
| 7 | Author's resume | NA |
