# Supplementary material for: Serum long noncoding RNA FAM83H-AS1 serves as a potential noninvasive diagnostic biomarker for ovarian cancer
Source: J Ovarian Res. 2026 Feb 3;19:83. doi: 10.1186/s13048-026-01995-1 (PMC12955189; doi:10.1186/s13048-026-01995-1)
Supplement: Supplementary file 2 — Supplementary Material 2. [file 13048_2026_1995_MOESM2_ESM.pdf]

吉林省肿瘤医院医学伦理委员会

伦理审查意见

批件号：202208-012-01（科研）

项目名称	FAM83H、IL-33 及 ST2 在卵巢癌患者外周血中的表达及临床意义		
申请科室	检验科		
项目负责人	于秀艳		
临床研究机构	吉林省肿瘤医院		
审 查 类 别	科研课题申报	审 查 方 式	会议审查
审 查 日 期	2022 年 8 月 9 日		
审 查 文 件	科研课题申报，详见附件		

审查意见：

依据《中华人民共和国执业医师法》、《医疗机构管理条例》、《药物临床试验质量管理规范》、《涉及人体的生物医学研究伦理审查办法》、《药物临床试验伦理审查工作指导原则》、世界医学会《赫尔辛基宣言》、世界卫生组织《生物医学研究审查伦理委员会操作指南》、国际医学科学组织委员会《涉及人的生物医学研究国际伦理准则》等法律、法规和国际准则，伦理委员会于 2022 年 8 月 9 日对上述审查文件进行了认真讨论并投票表决，结果如下：参会人数 11 人，投票人数 11 人，同意 11 票，必要的修改后同意 0 票，不同意 0 票。经本伦理委员会审查，审查结果决定为同意按照所批准的文件申报本项研究。

请遵循 GCP 原则、遵循伦理委员会批准的方案开展临床研究，保护受试者的健康与权利。

1. 研究获得审批后，请提交开展研究伦理审查；
2. 研究过程中若变更主要研究者，对临床研究方案、知情同意书、招募材料等的任何修改，请申请人提交修正案审查申请；
3. 发生可疑且非预期严重不良反应，请递交 SUSAR 审查申请；
4. 研究者没有遵从方案开展研究，可能对受试者的权益/健康、以及研究的科学性造成不良影响，请提交违背方案报告；
5. 申请人暂停或提前终止临床研究，请及时提交暂停/终止研究报告；
6. 研究结束时，请提交结题报告。

主任委员签字：	李慧	签字日期：	2022、8、10
伦理委员会公章：	吉林省肿瘤医院伦理委员会		

地点：长春市锦湖大路 1066 号，
电话：0431-80596067
邮编：130000

扫描全能王
3亿人都在用的扫描App

吉林省肿瘤医院伦理委员会参会委员名单

一、吉林省肿瘤医院伦理委员会声明:

吉林省肿瘤医院伦理委员会的职责、人员组成、操作程序及记录符合 ICH-GCP、中国 GCP 以及国家相关规定。伦理委员会成员将对伦理会议所审阅所有有关伦理审批资料以及伦理委员会会议的内容保密。

二、审核时间、地点:2022 年 08 月 08 日 腾讯网络视频会议，会议号:794-950-128

三、吉林省肿瘤医院伦理委员会成员名单及出席情况

姓　名	性 别	工作单位和技术职称	委员会职务	是否参会
李　慧	女	吉林省肿瘤医院研究员	主　任	参会
刘彦玲	女	吉林省肿瘤医院主任医师	副主任	参会
李光伟	女	吉林省肿瘤医院纪委书记	委　员	参会
景年财	男	吉林省肿瘤医院主任医师	委　员	参会
孙宝胜	男	吉林省肿瘤医院主任医师	委　员	参会
张彩霞	女	吉林省肿瘤医院主任医师	委　员	参会
沈志纲	男	吉林省肿瘤医院主管药师	委　员	参会
邢巨颖	女	吉林省肿瘤医院副主任护师	委　员	参会
张　爽	女	吉林省肿瘤医院副主任医师	委　员	参会
李　爽	女	社会人员	委　员	参会
朱　振	男	吉林大学法学院教授	委　员	参会

吉林省肿瘤医院伦理委员会

扫描全能王
3亿人都在用的扫描App

附件：

《FAM83H、IL-33 及 ST2 在卵巢癌患者外周血中的表达及临床意义》

文件名称	版本及日期
吉林省肿瘤医院伦理审查申请备案表	NA
吉林省肿瘤医院科研项目伦理初始审查申请表	NA
吉林省肿瘤医院临床研究项目审核表	NA
吉林省卫生健康科技能力提升项目申请书（任务书）	申请日期：2022 年 8 月 9 日
研究项目方案	NA
知情同意书	版本号：20220808，版本日期：2022.8.8
本中心主要研究者简历	NA

CS 扫描全能王
3亿人都在用的扫描App
